# The impact of constant antibiotic prophylaxis in children affected by spinal dysraphism performing clean intermittent catheterization: a 2-year monocentric retrospective analysis

**DOI:** 10.1007/s00381-021-05337-y

**Published:** 2021-09-14

**Authors:** Francesco Mariani, Emanuele Ausili, Margherita Zona, Giacomo Grotti, Antonietta Curatola, Antonio Gatto, Claudia Rendeli

**Affiliations:** 1 Dipartimento Di Scienze Della Salute Della Donna, Del bambino e Di Sanità Pubblica, Fondazione Policlinico Universitario A. Gemelli IRCCS, Largo A. Gemelli 8, 00168 Rome, Italy; 2 Unità Operativa Spina Bifida e Uropatie Malformative, Dipartimento Di Scienze Della Salute Della Donna, Del bambino e Di sanità pubblica, Fondazione Policlinico Universitario A. Gemelli IRCCS, Rome, Italy

**Keywords:** Bacterial resistance, Chemoprophylaxis, Child, Meningocele

## Abstract

**Introduction:**

Spinal dysraphism (SD) is a general term used to refer to developmental abnormalities of the spine that involves many clinical conditions including myelomeningocele (MMC). In these patients, neurogenic bladder (NB) is a common and predisposing factor for renal damage; the most frequently used approach to manage this situation is based on clean intermittent catheterization (CIC) and anticholinergic drugs. Urinary tract infections (UTIs) are a significant concern for these patients, and antibiotic prophylaxis is frequently used even if it is still a debated topic of literature. The purpose of this paper is to investigate the role and the real effectiveness of antibiotic prophylaxis in the reduction of incidence of UTIs in patients with spina bifida performing CIC.

**Methods:**

We collected data of all patients performing CIC, who did their last follow-up visit in the period between January 2019 and January 2021, followed at the children multidisciplinary Spina Bifida Center of A. Gemelli Hospital in Rome. Data collected included age at referral, gender, type of SD lesion, serum creatinine and cystatin C levels, the use of anticholinergic medications, antibiotic prophylaxis and type of prophylaxis (oral/endovesical), age of starting prophylaxis with its duration/adherence, number of CIC/day and its duration, episodes of UTIs in the 2 years prior to the last follow-up, and presence and grade of vesical-ureteric reflux (VUR) on cystourethrogram.

**Results:**

A total of 121 patients with SD performing CIC was included in the study; 66 (54%) presented ≥ 1 episode of UTIs in the last two years and 55 (46%) none. During the study period, 85 (70%) patients received antibiotic prophylaxis (ABP group) and 36 (30%) did not (NABP group): no statistically significative difference in terms of UTI development was observed between the two groups (*p* = 0.17). We also evaluated compliance to the therapy; 71 patients (59%) took antibiotic prophylaxis constantly (CABP group) and 50 (41%) did not do antibiotic prophylaxis constantly or did not do antibiotic prophylaxis at all (NCABP group): we observed a statistically significative difference in terms of UTIs with a 2.2 times higher risk of development at least one episode of UTIs in NCABP group.

**Conclusion:**

In conclusion, antibiotic prophylaxis performed constantly, without interruption, is associated with a lower risk of developing urinary tract infections and consequently to develop renal failure in adulthood.

## Introduction

Spinal dysraphism (SD) is a general term that includes the overall group of defects derived from a maldevelopment of the ecto-dermal, mesodermal, and neuroectodermal tissues [1]; these conditions are the result of failed closure of the embryonic neural tube during the fetal life. The commonest and severe form is myelomeningocele (MMC), a pathologic condition characterized by a dorsally opened spinal cord [2]; other conditions, such as myeloschisis, meningocele, lipomeningomyelocele, and tethered cord, may be usually less severe and are part of the closed type of SD.

Most of these children present Arnold Chiari malformation, paraplegia, neurogenic bowel and bladder, and, a part of them, hydrocephalus [3]. Furthermore, about 50% of them develop first urinary tract infections (UTIs) by 15 months of age and 44% have more than 5 UTI episodes by age of 15 years [4].

The goal of the management of neurogenic bladder (NB) in patients with SD is to preserve renal function and to have independent continence of bowel and bladder at a developmentally appropriate age [5]. The most frequently used approach to manage NB is based on clean intermittent catheterization (CIC) [6].

Anticholinergic drugs can also be used alone or in combination with CIC to preserve the renal function in the high-risk group [7]. However, UTIs are a common concern in individuals who perform CIC; in patients with SD, the routine use of antibiotic prophylaxis to prevent UTIs is controversial and guidelines on UTI management specific to SB populations are few. Among these, there is the Center for Disease Control and Prevention Urologic and Renal protocol that promotes the use of antibiotics only in patients with grade V reflux or a hostile bladder [8], suggesting a dose of 15 mg/kg amoxicillin orally once daily through age 2 months and daily trimethoprim/sulfamethoxazole or nitrofurantoin from that age onwards. On the contrary, there are some studies that discourage antibiotic prophylaxis, especially broad-spectrum one, for the onset of bacterial resistance [9–12], also seeing as how discontinuing low-dose chemoprophylaxis in patients with spina bifida on CIC does not significantly increase the number of febrile urinary tract infections [13].

The main aim of our study is to evaluate the efficacy of antibiotic prophylaxis to reduce the incidence of UTIs in patients with spina bifida treated with CIC.

## Methods

We performed a retrospective observational cross-sectional study on patients with spinal dysraphism (SD) followed at the Children’s multidisciplinary Spina Bifida center of Tertiary University Hospital “A. Gemelli” in Rome. Usually, children were evaluated with follow-up visits every year, and nephrological and urological medical histories were collected, creatinine, cystatin C levels, urinalysis, and culture by catheterized urine samples were performed. Patients performed a cystourethrogram (CUG) and when available these data were collected.

We included all patients treated with clean intermittent catheterization (CIC) during waking hours and those who performed their last follow-up visit between January 2019 and January 2021. Their clinical records were systematically reviewed and data collected included age at referral, gender, type of SD lesion (myelomeningocele or other, that included lipomeningomyelocele, tethered cord, and dermal sinus), serum creatinine and cystatin C levels, use of anticholinergic medications, antibiotic prophylaxis and type of prophylaxis (oral/in the bladder), age of starting prophylaxis and its duration, number of CIC/day, episodes of UTIs in the 2 years prior to the last follow-up, and presence and grade of vesical-ureteric reflux (VUR) on CUG.

Data about compliance to the prophylaxis were also collected; when two or more visits were performed in the last 2 years, if in both visits the patient was doing prophylaxis, he was considered adherent, while, if in even one was not doing prophylaxis, he was considered not adherent and the prophylaxis was considered not constant. We considered UTIs all those characterized by a positive urine test (presence of nitrites or two between leucocytes, esterase, hemoglobin, and proteins) and a positive urine culture > 100,000 CFU/mL [14].

For continuous variables, the Kolmogorov–Smirnov test was used to assess whether the distribution was normal or not. Categorical variables were reported as count and percentage. Continuous variables with normal distribution were expressed in terms of mean and standard deviation; data with non-normal distribution were expressed as median and interquartile range (25–75%). Statistical comparisons between groups were obtained by chi-squared tests for categorical variables and Mann–Whitney *U* test for continuous variables if not a normal distribution. *P* < 0.05 was considered statistically significant.

A logistic regression was performed to evaluate the possible impact of a constant antibiotic prophylaxis in the prevention of UTIs.

Statistical analysis was performed using IBM SPSS Statistics 23.0 software (IBM Corporation, Armonk, NY, USA).

## Results

We reviewed a total of 133 medical charts of patients affected by SD followed at the Children’s multidisciplinary Spina Bifida center of our hospital; 12 patients were excluded because their data regarding urine exams and urine culture were not available. A total of 121 patients were included in the study. The mean age of patients was 16.6 years (± 6.8 SD), 77 females and 46 males. Ninety patients (74.4%) were affected by MMC and 31 (25.6%) had other forms of SD such as dermal sinus, lipomeningomyelocele, and tethered cord.

All these patients performed CIC and the median age at the start of this treatment was 4.5 years (confidence interval 2.1–8.1); the median number of CIC per day was 4 (4–5) and 85.9% of these patients were treated with anticholinergic drugs.

As shown in Table 1 during the study period, 85 (70.2%) patients received antibiotic prophylaxis (ABP group) and 36 (30.8%) did not (NABP group). Antibiotic prophylaxis received by patients in the ABP group was oral in 61 cases (50.4%), with bladder instillation in 14 (11.5%) cases and both in 10 cases (8.2%). In addition, 71 children (58.7%) carried out prophylaxis in the last 2 years constantly and they were evaluated as constant antibiotic prophylaxis (CABP group) while the other 50 (41.3%) were included in the not-constant antibiotic prophylaxis (NCABP group); specifically, in this group, 36 patients did not receive antibiotic prophylaxis and 14 received antibiotic prophylaxis not constantly.

**Table 1:** Differences between non-antibiotic prophylaxis group (NABP group) and antibiotic prophylaxis group (ABP group)

	**NABP group** **(*n* 36)**	**ABP group** **(*n* 85)**	***p***
**Gender**			
Male	16 (44%)	29 (34%)	
Female	20 (56%)	56 (66%)	0.28
**Diagnosis**			
MMC	22 (61%)	68 (80%)	
Other	14 (39%)	17 (20%)	0.02
**Anticholinergic therapy**			
No	7 (19%)	10 (12%)	
Yes	29 (81%)	75 (88%)	0.26
Vesicoureteral reflux*			
No	32 (89%)	62 (74%)	
Yes	4 (11%)	22 (26%)	0.06
VUR > II grade*			
No	35 (97%)	77 (92%)	
Yes	1 (3%)	7 (8%)	0.43
**UTIs**			
< 1	13 (36%)	42 (49%)	
> / = 1	23 (64%)	43 (51%)	0.17

*A single patient did not perform cystography, so patients in which it was possible to evaluate VUR were 120

CUG was performed in 120 (99.2%) patients; 26 (21.6%) of them showed VUR, bilateral in 4 cases (15.4%) and higher than grade II in 8 cases (30.8%).

In the study population, we observed that 66 (54.5%) patients had more than 1 episode of UTIs in the last 2 years and 55 (46.5%) none.

Analyzing the two groups (NABP vs ABP), we identified a statistical difference correlated with the type of spinal dysraphism (*p* = 0.02); considering gender, anticholinergic therapy, VUR, and the presence of at least one episode of UTI in the last 2 years, no statistical difference was observed (Table 1).

In the ABP group, we found a median of UTIs of 0 (0–2); instead in the NABP group, we found a median of 1 (0–2) (*p* = 0.14).

Regarding serum creatinine, we collected data of 79 patients; a median of 0.85 mg/L (0.47–0.7) was observed in the ABP group and a median of 0.63 mg/L (0.55–0.83) in the NABP group (*p* = 0.27).

Cystatin level was collected in 69 patients; the mean value of serum cystatin C level was 0.84 ± 0.1 mg/L in the ABP group and 0.8 ± 0.01 mg/L in the NABP group (*p* = 0.8).

Afterwards, we compared patients who developed UTIs in the last 2 years and those who did not (Table 2). No statistically significant difference was noted in the duration of prophylaxis, duration of CIC, presence of VRU, grade of VUR higher than two, and number of CIC per day in the group with UTIs vs not UTIs.

**Table 2:** Differences between children with UTIs and children without UTIs

	**Urinary tract infections**		
	**Yes** **(*n* 66)**	**No** **(*n* 55)**	***p***
Vesicoureteral reflux*			
No	53 (81%)	41 (75%)	
Yes	12 (19%)	14 (25%)	0.35
Reflux > II grade*			
No	60 (92%)	52 (95%)	
Yes	5 (8%)	3 (5%)	0.7
**CIC (n/die)**	5 (4–5)	4 (4–5)	0.33
**CIC duration (y)**	10.5 (6.1)	10.3 (5.8)	0.9
**Constant prophylaxis**			
No	33 (50%)	17 (31%)	
Yes	33 (50%)	38 (69%)	0.03
**Prophylaxis duration (y)**	11.4 (± 7)	12.3 (± 5.9)	0.54

*A single patient did not perform cystography, so patients in which it was possible to evaluate VUR were 120

We also evaluated the impact of the different typologies of prophylaxis on the risk of UTI development. Between the patients who developed UTIs, 27 (63%) performed oral prophylaxis, 10 (23%) endovesical prophylaxis, and 6 (14%) both, while, between the patients who did not develop UTIs, 34 (80%) performed oral prophylaxis, 4 (10%) endovesical prophylaxis, and 4 (10%) both; no statistically significant difference was observed (*p* = 0.15).

Considering patients in the ABP group, we analyzed compliance to the therapy. We observed that 14 didn’t perform antibiotic prophylaxis constantly (they performed antibiotic prophylaxis for less than 12 months in the last 2 years), so we decided to divide the study population into other two groups: the first one involving patients who did antibiotic prophylaxis constantly (CABP group) and the second one involving children who did not assume antibiotic prophylaxis constantly or did not do antibiotic prophylaxis at all (NCABP group) (Table 3).

**Table 3:** Differences between not-constant antibiotic prophylaxis group (NCABP group) and constant antibiotic prophylaxis group (CABP group)

	**NCABP group** **(*n* 50)**	**CABP group** **(*n* 71)**	***p***
**Gender**			
Male	21 (42%)	24 (34%)	
Female	29 (58%)	47 (66%)	0.35
**Diagnosis**			
MMC	32 (64%)	58 (82%)	
Other	18 (36%)	13 (18%)	0.028
**Anticholinergic therapy**			
No	11 (22%)	6 (9%)	
Yes	39 (78%)	65 (91%)	0.035
Vesicoureteral reflux*			
No	41 (82%)	53 (76%)	
Yes	9 (18%)	17 (24%)	0.4
VUR > II grade*			
No	48 (96%)	64 (91%)	
Yes	2 (4%)	6 (9%)	0.3
**UTIs**			
< 1	17 (34%)	38 (54%)	
> / = 1	33 (66%)	33 (46%)	0.03

*A single patient did not perform cystography, so patients in which it was possible to evaluate VUR were 120

We observed statistically significant difference considering the incidence of UTIs in these two groups; in the CABP group, at least one UTI occurred in 33 (46%) patients and 38 (54%) did not develop UTIs, while in the NCABP group, at least one UTI occurred in 33 (66%) patients and 17 (34%) patients did not develop UTIs (*p* = 0.03).

A logistic regression was performed to assess whether constantly performed antibiotic prophylaxis could reduce the risk of urinary tract infections. The analysis showed that not performing antibiotic prophylaxis or not performing it constantly increases of 2.2 times the risk of developing at least one episode of UTIs (*p* < 0.05, IC 1.05–4.72).

Two other statistically significant differences were observed between the two groups. In the CABP group, MMC-affected patients were more represented (*p* = 0.028) and anticholinergic therapy was prescribed more frequently (*p* = 0.035).

## Discussion

SD is a complex and heterogeneous condition that requires a multidisciplinary management. Urological complications are one of the most important eventualities to face in patients with SD. Masini et al. [15] have also focused on this aspect highlighting a preserved sphincter function in about 55.6% of subjects with a repaired closed SB unlike those with a repaired open one in which the prevalence is 17.8%.

Failure to recognize and treat urinary tract infections can quickly lead to life-threatening conditions, whereas overtreatment contributes to antibiotic resistance [16].

The most frequently used approach to manage NB is based on clean intermittent catheterization (CIC) that is recommended for all infants with neurogenic bladder [5], however, there is no consensus among European centers in terms of protocols for UTI prevention, diagnosis, and treatment in children with NBSD [17].

Because of the absence of a clear guideline for the diagnosis of UTIs in children with NB performing CIC, our definition of UTI was made with the purpose of not overestimate the number of UTIs. In consideration of the high risk of asymptomatic bacteriuria in patients performing CIC, we defined diagnostic for UTI a urine sample with at least 100,000 CFU/mL of single germ and a urine test positive for nitrites or for two between the following: leucocytes, esterase, hemoglobin, and proteins.

The use of antibiotic prophylaxis in children with NBSD performing CIC to prevent UTIs is debated with no clear evidence [13, 18–21]. In our sample, we found no differences in the UTI incidence between the ABP group and NABP group, but we observed a significative reduction in the UTI risk in patients performing a constant antibiotic prophylaxis (CABP group). It was possible to estimate a 2.2 times higher risk of developing UTIs in patients not performing constant antibiotic prophylaxis (*p* < 0.05) than in those who did not perform it constantly or those who did not perform antibiotic prophylaxis at all (NCABP group); no prior study evaluated the difference between constant and not-constant antibiotic prophylaxis in children affected by SD performing CIC.

Our results, therefore, suggested that antibiotic prophylaxis, if performed constantly, can be associated with a lower risk of UTIs. According to our results, Pickard et al. [22], in a RCT conducted on adult patients performing CIC, demonstrated a reduced frequency of UTIs in patients performing antibiotic prophylaxis.

Zegers et al. [13], in a survey conducted in 2011, showed that antibiotic prophylaxis was associated with a lower incidence of UTIs, nevertheless underlining that it is not crucial to reduce the risk of febrile UTIs. Our data collection did not allow us to evaluate how many UTI episodes were febrile; however, the stringent parameters (positive urinalysis and CFU of a single germ ≥ 100,000 CFU/mL) used in this study allowed us to consider with high probability that all forms of asymptomatic bacteriuria have been excluded and, therefore, to consider all reported infections as real.

No statistical differences in terms of duration of prophylaxis, presence of reflux, and type of prophylaxis were observed.

The absence of statistically significative differences in the duration of prophylaxis suggested a not harmful role of antibiotic prophylaxis in these patients, while the absence of correlation with the presence of reflux may be explained by the evidence that only the highest grades of reflux, poorly represented in our sample, are associated with an augmented risk of UTIs [23].

Duration of CIC seems to have no impact on UTI risk, allowing us to state that early starting of CIC is not associated with an increased risk of infection.

The absence of a statistically significant difference of cystatin and creatinine levels in the two groups (ABP e NABP) could indicate that prophylaxis does not lead to an improvement of these indices; however, it should be highlighted that the high number of missing observations can make this result scarcely reliable.

In conclusion, with the limits of a retrospective analysis, we can assert that an antibiotic prophylaxis performed constantly, without interruption, is associated with a lower risk of developing urinary tract infections. No other clear risk factors have been found, suggesting that even the duration of antibiotic prophylaxis is not associated with an increased risk of infection. Further prospective studies are needed to evaluate the impact of a constant antibiotic prophylaxis on UTI prevention in children affected by SD performing clean intermittent catheterization.

## Data Availability

The datasets used and/or analyzed during the current study are available from the corresponding author on reasonable request.
